# Combined treatment with a selective PDE10A inhibitor TAK‐063 and either haloperidol or olanzapine at subeffective doses produces potent antipsychotic‐like effects without affecting plasma prolactin levels and cataleptic responses in rodents

**DOI:** 10.1002/prp2.372

**Published:** 2017-12-18

**Authors:** Kazunori Suzuki, Akina Harada, Hirobumi Suzuki, Clizia Capuani, Annarosa Ugolini, Mauro Corsi, Haruhide Kimura

**Affiliations:** ^1^ CNS Drug Discovery Unit, Research Takeda Pharmaceutical Company Limited Fujisawa Kanagawa Japan; ^2^ Center for Drug Design & Discovery Aptuit Inc. Verona Italy

**Keywords:** antipsychotics, medium spiny neuron, phosphodiesterase 10A, schizophrenia, TAK‐063

## Abstract

Activation of indirect pathway medium spiny neurons (MSNs) via promotion of cAMP production is the principal mechanism of action of current antipsychotics with dopamine D_2_ receptor antagonism. TAK‐063 [1‐[2‐fluoro‐4‐(1H‐pyrazol‐1‐yl)phenyl]‐5‐methoxy‐3‐(1‐phenyl‐1H‐pyrazol‐5‐yl)pyridazin‐4(1H)‐one] is a novel phosphodiesterase 10A inhibitor that activates both direct and indirect pathway MSNs through increasing both cAMP and cGMP levels by inhibition of their degradation. The activation of indirect pathway MSNs through the distinct mechanism of action of these drugs raises the possibility of augmented pharmacological effects by combination therapy. In this study, we evaluated the potential of combination therapy with TAK‐063 and current antipsychotics, such as haloperidol or olanzapine after oral administration. Combined treatment with TAK‐063 and either haloperidol or olanzapine produced a significant increase in phosphorylation of glutamate receptor subunit 1 in the rat striatum. An electrophysiological study using rat corticostriatal slices showed that TAK‐063 enhanced *N*‐methyl‐_D_‐aspartic acid receptor‐mediated synaptic responses in both direct and indirect pathway MSNs to a similar extent. Further evaluation using pathway‐specific markers revealed that coadministration of TAK‐063 with haloperidol or olanzapine additively activated the indirect pathway, but not the direct pathway. Combined treatment with TAK‐063 and either haloperidol or olanzapine at subeffective doses produced significant effects on methamphetamine‐ or MK‐801‐induced hyperactivity in rats and MK‐801‐induced deficits in prepulse inhibition in mice. TAK‐063 at 0.1 mg/kg did not affect plasma prolactin levels and cataleptic response from antipsychotics in rats. Thus, TAK‐063 may produce augmented antipsychotic‐like activities in combination with antipsychotics without effects on plasma prolactin levels and cataleptic responses in rodents.

**Abbreviations**
AMPA: (±)‐α‐amino‐3‐hydroxy‐5‐methylisoxazole‐4‐proprionic acid
ANOVA: analysis of variance
Compound 1: [1‐[cyclopropylmethyl]‐4‐fluoro‐5‐[5‐methoxy‐4‐oxo‐3‐(1‐phenyl‐1H‐pyrazol‐5‐yl)pyridazin‐1(4H)‐yl]‐3,3‐dimethyl‐1,3‐dihydro‐2H‐indol‐2‐one]
EPSC: excitatory postsynaptic current
EPS: extrapyramidal symptoms
EPSP: excitatory postsynaptic potential
GABA: gamma aminobutyric acid
GluR1: glutamate receptor subunit 1
METH: methamphetamine
MK‐801: [(5*R*,10*S*)‐(+)‐5‐methyl‐10,11‐dihydro‐5*H*‐dibenzo[a,d]cyclohepten‐5,10‐imine]
MSN: medium spiny neuron
NBQX: 1,2,3,4‐tetrahydro‐6‐nitro‐2,3‐dioxo‐benzo[f]quinoxaline‐7‐sulfonamide
NMDA: *N*‐methyl‐_D_‐aspartate
PCR: polymerase chain reaction
PDE: phosphodiesterase
TAK‐063: [1‐[2‐fluoro‐4‐(1*H*‐pyrazol‐1‐yl)phenyl]‐5‐methoxy‐3‐(1‐phenyl‐1*H*‐pyrazol‐5‐yl)pyridazin‐4(1*H*)‐one]

## 1 INTRODUCTION

Dopamine D_2_ receptor antagonism or partial agonism is the principal mechanism of action of current antipsychotics.^1^, ^2^, ^3^, ^4^ D_2_ receptors are predominantly expressed in striatal indirect pathway medium spiny neurons (MSNs) that are coupled to an inhibitory G protein (G_i_), which inhibits the activity of adenylate cyclase. This enzyme synthesizes cAMP from ATP^5^; thus, current antipsychotics with D_2_ antagonism or partial agonism are thought to produce antipsychotic effects via activation of the indirect pathway by promoting cAMP production. However, approximately 30% of patients with schizophrenia receive little or no benefit from current antipsychotics,^6^, ^7^, ^8^ and the use of typical antipsychotics can be associated with mechanism‐related side effects such as extrapyramidal symptoms (EPS) and hyperprolactinemia.^9^ Even atypical antipsychotics with a lower incidence of EPS than typical antipsychotics are still associated with hyperprolactinemia and serious metabolic side effects.^9^ Thus, a new therapeutic option with improved antipsychotic efficacy and a better side effect profile would provide a significant advancement in the treatment of schizophrenia.

Phosphodiesterase 10A (PDE10A), a dual‐substrate PDE that hydrolyzes both cAMP and cGMP, is highly expressed in both direct and indirect pathway MSNs.^10^, ^11^, ^12^ Therefore, the inhibition of PDE10A increases both cAMP and cGMP levels by inhibiting their degradation, thereby enhancing downstream signal transduction in both direct and indirect MSN pathways. TAK‐063 [1‐[2‐fluoro‐4‐(1H‐pyrazol‐1‐yl)phenyl]‐5‐methoxy‐3‐(1‐phenyl‐1H‐pyrazol‐5‐yl)‐pyridazin‐4(1H)‐one] is a selective PDE10A inhibitor.^13^, ^14^ Compared with other PDE10A inhibitors such as MP‐10 and compound 1 [1‐[cyclopropylmethyl]‐4‐fluoro‐5‐[5‐methoxy‐4‐oxo‐3‐(1‐phenyl‐1H‐pyrazol‐5‐yl)pyridazin‐1(4H)‐yl]‐3,3‐dimethyl‐1,3‐dihydro‐2H‐indol‐2‐one] (previously synthesized by Takeda Pharmaceutical Limited,^15^ TAK‐063 showed comparable activation of indirect pathway MSNs while producing partial activation of direct pathway MSNs by its fast dissociation property in the rat striatum.^15^ These MSN pathways have competing effects on antipsychotic‐like activities and motor functions in rodents.^15^, ^16^, ^17^ TAK‐063 with this unique activation pattern of MSN pathways produces antipsychotic‐like effects in the multiple paradigms of rodent models.^15^ TAK‐063 also has a lower risk of inducing a cataleptic response than haloperidol, olanzapine, and aripiprazole in rats.^17^ PDE10A inhibition by TAK‐063 could be a novel therapeutic approach to treating schizophrenia. Current antipsychotics with D_2_ antagonism only activate the indirect pathway, whereas TAK‐063 can activate both direct and indirect pathways.^15^, ^17^ Combination therapy with TAK‐063 and current antipsychotics raises the possibility to augment the pharmacological efficacy by further activation of indirect pathway MSNs while maintaining the activation of direct pathway MSNs. The pharmacological profile of the combination of TAK‐063 and current antipsychotics remains to be evaluated.

In this study, we evaluated the effects of TAK‐063 combined with current antipsychotics haloperidol or olanzapine on the activation of MSN pathways, antipsychotic‐like effects, plasma prolactin levels, and cataleptic responses in rodents. Here, we report preclinical evidence that TAK‐063 may be beneficial as combination therapy with current antipsychotics without effects on plasma prolactin levels and cataleptic responses, which may be indicative of propensity to cause EPS such as drug‐induced Parkinsonism.^18^

## 2 MATERIALS AND METHODS

### 2.1 Animals

Male institute of cancer research (ICR) mice and Sprague‐Dawley (SD) rats were supplied by CLEA Japan, Inc. (Tokyo, Japan) and Charles River Laboratories Japan Inc. (Yokohama, Japan), respectively. Animals were housed in a light‐controlled room (12‐h light/12‐h dark cycle with lights on at 07:00 am). After an acclimation period of at least 1 week, 6‐ to 9‐week‐old animals were used. The care and use of the animals and the experimental protocols were approved by the Institutional Animal Care and Use Committee of Takeda Pharmaceutical Company Limited (Osaka and Fujisawa, Japan). Four‐week‐old male SD rats were supplied by Charles River Laboratories Italia (Italy) for the electrophysiological study which was conducted by Aptuit Inc. (Verona, Italy) in accordance with national legislation and under authorization issued by the Italian Ministry of Health. Aptuit Inc. is committed to the highest standards of animal welfare and is subject to legislation under the Italian Legislative Decree no. 26/2014.

### 2.2 Drugs

TAK‐063 was synthesized by Takeda Pharmaceutical Company Limited. Haloperidol was purchased from Sigma‐Aldrich Co. LLC. (St. Louis, MO). Olanzapine was extracted from Zyprexa^®^ (Eli Lilly and Company, Indianapolis, IN) at KNC Laboratories Co. Ltd. (Kobe, Japan) or purchased from AK Scientific Inc. (Union City, CA). Aripiprazole was purchased from AK Scientific Inc. TAK‐063 and haloperidol were suspended in 0.5% (w/v) methylcellulose in distilled water and administered orally (p.o.). Olanzapine was dissolved in 1.5% (v/v) lactic acid. The pH of this solution was then adjusted to neutral using 1 mol L^−1^ NaOH and administered orally. Aripiprazole was suspended in a 1% (v/v) solution of Tween 80 in distilled water and administered orally. Methamphetamine hydrochloride (METH, Dainippon Sumitomo Pharma Co. Ltd., Osaka, Japan) and (+)‐MK‐801 hydrogen maleate (MK‐801, Sigma‐Aldrich) were dissolved in saline and administered subcutaneously (s.c.). All compounds were dosed at 20 and 2 mL/kg body weight in mice and rats, respectively.

### 2.3 Measurement of phosphorylated GluR1 level in the rat striatum

TAK‐063 and haloperidol were coadministered to male SD rats 2 hours before sacrifice. TAK‐063 and olanzapine were administered to male SD rats 2 and 0.5 hours, respectively, before sacrifice. Pretreatment times were determined according to the pharmacokinetic profile (ie, time to maximum plasma concentration, Tmax) of each agent. Following sacrifice, striatum was isolated and homogenized in extraction buffer (Invitrogen, Carlsbad, CA) containing 0.5 mmol L^−1^ p‐amidinophenylmethanesulfonyl fluoride hydrochloride (Sigma‐Aldrich) and the protease inhibitor cocktail (Complete Mini, Roche Applied Science, Indianapolis, IN). The homogenates were centrifuged at 20400g for 10 minutes at 4°C. After centrifugation, protein concentrations in the supernatant were measured using the BCA protein assay kit (Thermo Fisher Scientific, Waltham, MA). Each sample (containing 10 μg protein) was subjected to electrophoresis though polyacrylamide gels, followed by Western blotting to polyvinylidene difluoride membranes (Bio‐Rad Laboratories Inc., Hercules, CA). The blots were incubated with antibodies against phosphorylated (±)‐α‐amino‐3‐hydroxy‐5‐methylisoxazole‐4‐proprionic acid (AMPA) receptor subunit 1 (GluR1) at Ser845 (pGluR1, PhosphoSolutions^®^, Aurora, CO) or total GluR1 (Millipore, Temecula, CA). Individual protein bands were visualized with secondary antibodies conjugated to horseradish peroxidase (GE Healthcare UK Ltd., Buckinghamshire, UK) followed by treatment with ECL Prime Western Blotting Detection Reagent (GE Healthcare UK Ltd.) or ImmunoStar LD (Wako Pure Chemical Industries, Osaka, Japan). The chemiluminescent signals were detected by an ImageQuant LAS 4000 imaging system (GE Healthcare UK Ltd.), and densitometric analysis was performed using ImageQuant TL Software (GE Healthcare UK Ltd.). Signal densities of phosphorylated protein bands for each sample were normalized to the signal density of the corresponding total protein bands. These ratios are expressed as the relative change divided by the average of the vehicle‐treated samples.

### 2.4 Measurement of NMDA receptor responses in direct and indirect pathway MSNs in rat corticostriatal slices

This experiment was conducted in Aptuit Inc. Four‐week‐old male SD rats were anesthetized with isoflurane and decapitated. The brain was quickly removed and placed in an oxygenated, ice‐cold solution containing NaCl (125 mmol L^−1^), KCl (2.5 mmol L^−1^), CaCl_2_ (2 mmol L^−1^), MgCl_2_ (1 mmol L^−1^), glucose (25 mmol L^−1^), pH 7.4, saturated with 95% O_2_ and 5% CO_2_. Acute parasagittal corticostriatal slices (300 μm) were incubated in an extracellular recording solution containing NaCl (125 mmol L^−1^), KCl (2.5 mmol L^−1^), NaH_2_PO_4_ (1.25 mmol L^−1^), CaCl_2_ (2 mmol L^−1^), NaHCO_3_ (25 mmol L^−1^), and glucose (25 mmol L^−1^), saturated with 95% O_2_ and 5% CO_2_, at 32°C at least 1 hour before recording. Whole‐cell recordings were performed on individual MSNs visualized by infrared differential interference contrast microscopy and identified by a typical repetitive and nonadapting spike‐firing pattern in response to depolarizing current step injection and a rectification during hyperpolarizing steps.^19^ The two types of neurons (direct pathway and indirect pathway MSNs) were distinguished by their different membrane properties and suprathreshold corticostriatal response evoked by extracellular stimulation.^20^, ^21^, ^22^, ^23^ In our conditions, the rheobase current was significantly greater in direct pathway MSNs than in indirect pathway MSNs (direct pathway MSNs: median, 400 pA, n = 70; indirect pathway MSNs: median, 175 pA, n = 52; *P *<* *.001 by unpaired *t* test). There was a significant difference in input resistance measured with a ‐150 pA hyperpolarizing step from the resting membrane potential (direct pathway MSNs: median, 37 MΩ, n = 70; indirect pathway MSNs: median, 80 MΩ, n = 52; *P *<* *.001 by unpaired t‐test). The differences in rheobase were not reflective of differences in spike threshold (direct pathway MSNs: median, −49 mV, n = 70; indirect pathway MSNs: median, −50 mV, n = 52; *P *>* *.05 by unpaired t‐test) and also the resting membrane potential was similar in the two types of MSNs (direct pathway MSNs: median, −73 mV, n = 70; indirect pathway MSNs: median, −71 mV, n = 52; *P *>* *.05 by unpaired *t* test). The suprathreshold evoked response of indirect pathway MSNs is characterized by a prolonged train of action potentials followed by a quasi‐exponential decay while that of direct pathway MSNs is characterized by an initial burst of action potentials, followed by a decaying plateau.^20^ Recordings were carried out using whole‐cell patch pipette filled with a solution containing K‐gluconate (114 mmol L^−1^), KCl (6 mmol L^−1^), MgATP (4 mmol L^−1^), NaGTP (0.3 mmol L^−1^), Na‐Phosphocreatine (10 mmol L^−1^), HEPES (10 mmol L^−1^), EGTA (0.2 mmol L^−1^), pH 7.25 with KOH, osmolarity 300 mOsm with sucrose. Electrical signals were recorded through a Multiclamp 700B patch‐clamp amplifier and digitized using a Digidata 1550B interface and pClamp 10.6 software (Molecular Devices LLC, Sunnyvale, CA); records were acquired at a sampling rate of 10 kHz and filtered at 10 kHz. The liquid junction potential (LJP) was −12 mV and should be added to all voltages to obtain the correct membrane potentials.^24^ The evoked excitatory postsynaptic current (EPSC, recorded holding neurons close to the resting potential at ‐80 mV, after LJP correction) and excitatory postsynaptic potential (EPSP, recorded at resting potential without current injection) were evoked in neurons with stimulus intensity set at 50% of maximum response and duration of 100 μs, delivered every 15 seconds through a concentric bipolar tungsten electrode (TM33CCINS, World Precision Instruments, Sarasota, FL) placed in the corpus callosum and close to the recording electrode. The evoked EPSPs and EPSCs were recorded from dorsal striatum of different slice groups. *N*‐methyl‐_D_‐aspartate (NMDA) receptor‐mediated responses were isolated by the addition of NBQX (10 μmol L^−1^) and bicuculine (20 μmol L^−1^) to the extracellular solution; these compounds are selective antagonists of AMPA and gamma aminobutyric acid (GABA) A receptors, respectively. The PSP peak amplitudes and durations were analyzed using Clampfit 10.6 software. The peak amplitude was measured from the start of the rising phase to the peak depolarization, while the duration was measured as the 90%‐10% decay time determined from 90% of the peak of the PSP to 10% above baseline. As a control experiment, peak amplitudes and decay kinetics of EPSPs and EPSCs were evaluated in the presence of the vehicle (0.1% dimethyl sulfoxide). In testing drug effects, control responses were recorded, and then slices were perfused with 1 μmol L^−1^ of TAK‐063 for at least 15 minutes and EPSP and EPSC peak amplitudes and decay kinetics were measured at steady state.

### 2.5 Real‐Time quantitative polymerase chain reaction expression analysis of substance P and enkephalin mRNAs

Total RNA was extracted from the rat striatum using Isogen (Nippon Gene Co. Ltd., Toyama, Japan) and RNeasy kit (Qiagen, Hilden, Germany) according to manufacturers’ instructions. Real‐time quantitative polymerase chain reaction (PCR) was performed using an ABI PRISM 7900HT sequence detection system (Thermo Fisher Scientific Inc., Waltham, MA) and TaqMan reagents (Eurogentec, Seraing, Belgium). The expression levels of substance P and enkephalin mRNAs were normalized using glyceraldehyde‐3‐phosphate dehydrogenase TaqMan probes according to manufacturer's instruction. The following primers were used for substance P analysis (GenBank M34184): TaqMan probe, 5′‐CGTGGCGGTGGCGGTCTTTTT‐3′; forward primer, 5′‐CGCAAAATCCAACATGAAAATC‐3′; reverse primer, 5′‐GCAAACAGTTGAGTGGAAACGA‐3′. The following primers were used for enkephalin analysis (GenBank M28263): TaqMan probe, 5′‐CGCCTGGTACGTCCCGGCG‐3′; forward primer, 5′‐GGACTGCGCTAAATGCAGCTA‐3′; reverse primer, 5′‐GTGTGCATGCCAGGAAGTTG‐3′.

### 2.6 METH‐ or MK‐801‐induced hyperactivity in rats

Locomotor activity was measured using a SUPERMEX spontaneous motor activity analyzer (Muromachi Kikai Co. Ltd., Tokyo, Japan). Male SD rats were placed in locomotor chambers (length × width × height: 24 × 37 × 30 cm) for more than 1 hour for habituation. The rats were then removed from each chamber and treated with either vehicle or test compounds and quickly returned to the chamber. After an appropriate pretreatment time, the rats were again removed from the chambers and treated with either vehicle (saline), METH (0.5 mg/kg s.c., as a salt), or MK‐801 (0.3 mg/kg s.c., as a salt) and then quickly returned to the test chamber. Pretreatment times varied according the pharmacokinetic profile (ie, Tmax) of each agent. Test compounds were administered to METH treatment groups in a blind manner. Activity counts were recorded in successive 1‐minute bins and then cumulative counts during the 2‐hour period after psychostimulant administrations were calculated.

### 2.7 Prepulse Inhibition of an acoustic startle response in mice

Test compounds and MK‐801 (0.3 mg/kg s.c., as a salt) were administered to male ICR mice 60 minutes and 20 minutes, respectively, before testing in a blind manner. Pretreatment time was determined according to the pharmacokinetic profile (Tmax) of TAK‐063 (Figure S1). Test sessions consisted of placement of individual animals into the startle chamber and initiation of the background noise (70 db). After a 5‐minute acclimation period, each subject was presented with 54 trials with variable intertrial intervals (7‐23 seconds). Three types of trials were used: (1) a pulse‐only trial of 118 or 82 db presented for 40 milliseconds during which the startle response was recorded for 40 milliseconds beginning with the onset of a pulse; (2) prepulse trials consisting of 118 db presented for 40 milliseconds, preceded 100 milliseconds earlier by a 20‐millisecond prepulse of 76 or 82 db during which the startle response was recorded for 40 milliseconds beginning with the onset of 118 db; and (3) a no‐stimulus trial in which only the background noise was present. Mice with an average maximum startle amplitude of ≤25 units on pulse‐only trials at 118 db were excluded from the data analyses. The percentage of prepulse inhibition (PPI) of the 82‐db prepulse was calculated using the following formula: [(average maximum startle on pulse‐only trials at 118 db − average maximum startle on prepulse trials)/average maximum startle on pulse‐only trials at 118 db × 100].

### 2.8 Measurement of plasma prolactin levels

After a habituation to the behavioral test room environment (>30 minutes), male SD rats were orally coadministered either vehicle or test compounds. Ninety minutes after coadministration, blood was collected from tail veins into a 1.5‐mL tube containing 25 μL of EDTA. Blood was immediately mixed with EDTA, and then centrifuged at 13000g for 15 minutes at 4°C. The supernatant was collected into another tube as plasma and was stored in a deep freezer until use. The prolactin concentrations in these plasma samples were determined using an ELISA kit (Bertin Pharma, Montigny le Bretonneux, France).

### 2.9 Bar test

The degree of cataleptic response in rats by antipsychotics was reported to increase as a function of both time and dose.^25^ The cataleptic behavior of male SD rats was measured 4 hours after coadministration of test compounds in a blind manner. Forelimbs were placed on a horizontal metal bar at 13‐cm height and the length of time both forelimbs remained on the bar was measured using a stopwatch (Seiko Holdings Corporation, Tokyo, Japan). Rats remaining on the bar for ≥90 seconds were assigned a latency of 90 seconds (cutoff value). The average of three trials was calculated as the duration of the cataleptic response.

### 2.10 Statistical analysis

Aspin‐Welch tests were carried out to assess the statistical significance of differences between two groups. Treatment effects of test compounds and their interactions were analyzed by two‐way analysis of variance (ANOVA). In electrophysiological study, differences between the control responses and effects of TAK‐063 were analyzed by a Wilcoxon matched‐pairs signed rank test. Differences yielding *P* values ≤ .05 were considered significant.

## 3 RESULTS

### 3.1 Combined treatment with TAK‐063 and either haloperidol or olanzapine significantly increased in pGluR1 in the rat striatum

**Figure 1:**
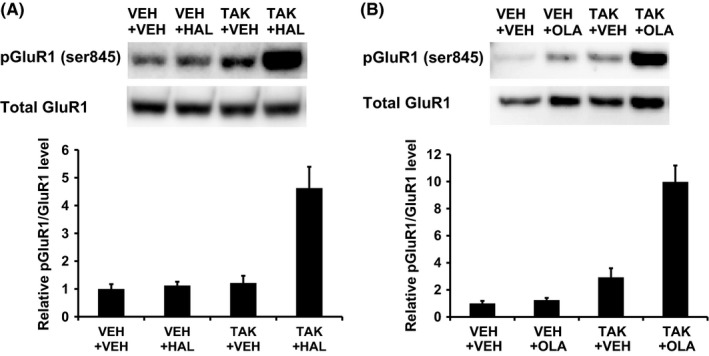
The effect of TAK‐063 combined with either haloperidol or olanzapine on pGluR1 in the rat striatum. pGluR1 levels were measured by Western blot analysis. Representative Western blot images are shown. Densities of pGluR1 bands for each sample were normalized to the corresponding GluR1 band density. Data are represented as mean + SEM (n = 4). (A) The striatum was dissected 2 hours after coadministration of TAK‐063 (TAK, 0.3 mg/kg p.o.) and haloperidol (HAL, 0.3 mg/kg p.o.). The two‐way ANOVA showed a significant interaction between TAK and HAL (*F*_1,12_ = 15.55; *P *≤* *.05). (B) The striatum was dissected 2 and 0.5 hours after administration of TAK‐063 (TAK, 0.3 mg/kg p.o.) and olanzapine (OLA, 3 mg/kg p.o.), respectively. The two‐way ANOVA showed a significant interaction between TAK and OLA (*F*_1,12_ = 23.56; *P *≤* *.05)

Given the high level of expression of PDE10A in MSNs, PDE10A inhibition and the resulting elevation of cAMP and cGMP increased the phosphorylation of their downstream target molecules, such as AMPA receptor GluR1 subunit.^10^, ^26^, ^27^, ^28^, ^29^ To assess the activation of downstream cAMP signaling by the combination of a PDE10A inhibitor TAK‐063 and either haloperidol or olanzapine, pGluR1 in the rat striatum was evaluated by Western blot analysis. PDE10A inhibition by TAK‐063 at 0.3 mg/kg or more dose‐dependently upregulates cAMP and cGMP levels, activates their downstream signaling in the striatum, and produces potent antipsychotic‐like effects evaluated by METH‐ or MK‐801‐induced hyperactivity rodent models of psychosis.^15^, ^17^ Haloperidol at 0.3 mg/kg p.o. or olanzapine at 3 mg/kg p.o. also produced antipsychotic‐like effects in METH‐induced hyperactivity in rats.^15^ Thus, these dosages were used for the evaluation of pGluR1 in the rat striatum. TAK‐063 or haloperidol alone slightly increased pGluR1, whereas the combination of these two drugs induced a robust increase (Figure 1A). The two‐way ANOVA analysis showed a significant interaction between haloperidol and TAK‐063 (*F*_1,12_ = 15.55; *P ≤ *.05; Figure 1A). A significant interaction was also observed when TAK‐063 was combined with olanzapine (*F*_1,12_ = 23.56; *P ≤ *.05; Figure 1B).

### 3.2 Combined treatment with TAK‐063 and either haloperidol or olanzapine additively activated indirect pathway MSNs, but not direct pathway MSNs, in rats

**Figure 2:**
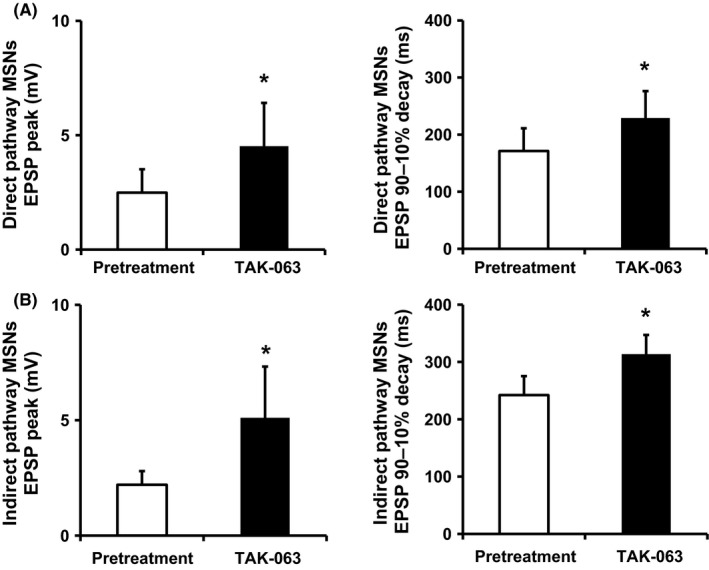
The effects of TAK‐063 on NMDA receptor‐mediated synaptic responses in direct and indirect pathway MSNs in rat corticostriatal brain slices. TAK‐063 at 1 μmol L^−1^ significantly increased peak amplitudes and decay times of NMDAR EPSP in direct (A) and indirect (B) pathway MSNs. The average of peak amplitudes and decay times during the 3 minutes before and after 20 minutes of compound application were calculated. Data are represented as mean + SEM (n = 7). *P ≤* *.05 vs control (pretreatment) by Wilcoxon‐matched pairs signed rank test

Striatal outputs mediated by MSNs are mainly divided into two pathways: D_1_ receptor‐expressing direct pathway and D_2_ receptor‐expressing indirect pathway.^30^, ^31^ Because PDE10A is highly expressed in both direct and indirect pathway MSNs, PDE10A inhibition is expected to increase neural activity in both types of MSNs thorough the activation of cAMP and cGMP signaling. To confirm enhancement of neural activities of each type of MSNs by TAK‐063, we evaluated the effects of TAK‐063 on NMDA receptor‐mediated synaptic responses in each type of MSNs using rat corticostriatal brain slices. Vehicle (0.1% dimethyl sulfoxide) did not change peak amplitudes or decay times of NMDAR EPSP and EPSC in MSNs for 30 minutes after application, indicating that vehicle had no effects on basal responses (Figure S2). Under these experimental conditions, TAK‐063 at 1 μmol L^−1^ significantly increased peak amplitudes and decay times of NMDAR EPSPs in both types of MSNs (Figure 2A and B) and the magnitude induced by TAK‐063 was similar in the direct and indirect pathway MSNs. Similar results were observed when peak amplitudes and decay times of NMDAR EPSCs were measured (Figure S3).

**Figure 3:**
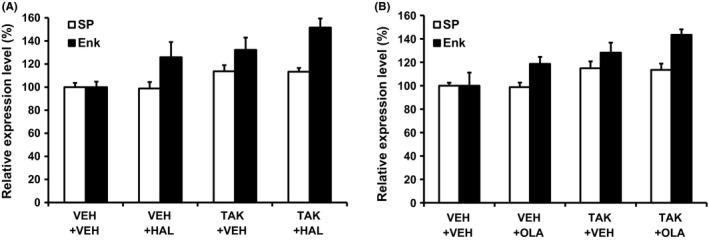
The effects of TAK‐063 coadministration with either haloperidol or olanzapine on the activation of direct and indirect pathway MSNs in the rat striatum. (A) Three hours after coadministration of TAK‐063 (TAK, 0.3 mg/kg p.o.) and haloperidol (HAL, 1 mg/kg p.o.), mRNA expression levels of substance P (SP, a marker of the direct pathway) and enkephalin (Enk, a marker of the indirect pathway) in the rat striatum were measured by PCR analysis. The values in the graph represent expression levels relative to that of the vehicle (VEH)‐treated group. Data are presented as mean + S.E.M. (n = 6). The two‐way ANOVA showed significant effects of HAL and TAK on Enk expression (HAL:* F*_1,20_ = 5.52; *P ≤ *.05; TAK:* F*_1,20_ = 9.13; *P ≤ *.05) and a significant effect of TAK on SP expression (*F*_1,20_ = 9.49; *P ≤ *.05). (B) Three hours after coadministration of TAK‐063 (TAK, 0.3 mg/kg p.o.) and olanzapine (OLA, 10 mg/kg p.o.), mRNA expression levels of SP and Enk in the rat striatum were measured. The values in the graph represent expression levels relative to those of the VEH‐treated group. Data are presented as mean + SEM (n = 6). The two‐way ANOVA showed significant effects of OLA and TAK on Enk expression (OLA:* F*_1,20_ = 4.52; *P ≤ *.05; TAK:* F*_1,20_ = 11.07; *P ≤ *.05) and a significant effect of TAK on SP expression (*F*_1,20_ = 10.50; *P ≤ *.05)

To further investigate the activation of MSNs by the combination of TAK‐063 and either haloperidol or olanzapine in vivo, we evaluated activation pattern of direct and indirect pathway MSNs in rats using induction of genes as pathway‐specific markers: substance P for the direct pathway and enkephalin for the indirect pathway.^32^, ^33^ Haloperidol at 1 mg/kg p.o. alone selectively increased enkephalin mRNA levels in the rat striatum (Figure 3A). TAK‐063 at 0.3 mg/kg p.o. increased both enkephalin and substance P mRNA levels in the rat striatum (Figure 3A). The combination of these drugs produced an increase in enkephalin mRNA levels, but not substance P mRNA levels (Figure 3A). The two‐way ANOVA showed significant effects of haloperidol and TAK‐063 on enkephalin expression (haloperidol: *F*_1,20_ = 5.52; *P ≤ *.05; TAK‐063: *F*_1,20_ = 9.13; *P ≤ *.05; Figure 3A) and a significant effect of TAK‐063 on substance P expression (*F*_1,20_ = 9.49; *P ≤ *.05; Figure 3A), although there was no significant interaction between haloperidol and TAK‐063. A similar result was observed when TAK‐063 at 0.3 mg/kg p.o. was combined with olanzapine at 10 mg/kg p.o.; the two‐way ANOVA analysis showed significant effects of olanzapine and TAK‐063 on enkephalin expression (olanzapine: *F*_1,20_ = 4.52; *P ≤ *.05; TAK‐063: *F*_1,20_ = 11.07; *P ≤ *.05; Figure 3A) and a significant effect of TAK‐063 on substance P expression (*F*_1,20_ = 10.50; *P ≤ *.05; Figure 3B), although there was no significant interaction between olanzapine and TAK‐063. These results suggest that combination of TAK‐063 and current antipsychotics additively increase the activation of indirect but not direct pathway MSNs, in the rat striatum.

### 3.3 Combined treatment with TAK‐063 and either haloperidol or olanzapine at subeffective doses has significant effects on METH‐ or MK‐801‐induced hyperactivity in rats

**Figure 4:**
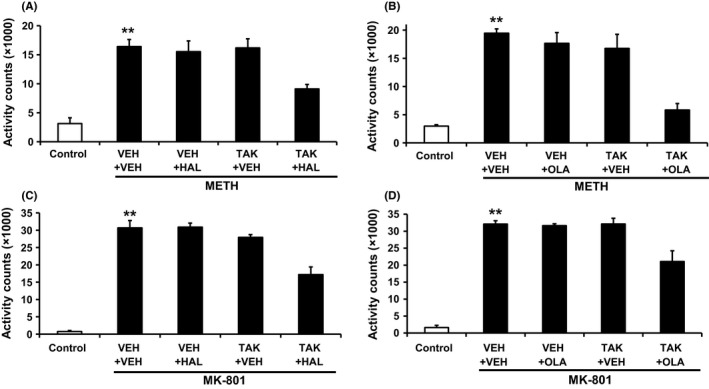
The effects of TAK‐063 combined with either haloperidol or olanzapine on METH‐ or MK‐801‐induced hyperactivity. (A) TAK‐063 (TAK, 0.1 mg/kg p.o.) and haloperidol (HAL, 0.2 mg/kg p.o.) were coadministered to rats 1.5 hours before treatment with METH (0.5 mg/kg s.c.). Accumulated activity counts during 2 hours after METH treatment were calculated and indicated as the mean + SEM (n = 6 in control group, n = 6 in vehicle (VEH) + METH group, n = 7 in TAK + METH group, n = 6 in HAL + METH group, n = 7 in TAK + HAL + METH group). The two‐way ANOVA showed a significant interaction between HAL and TAK (*F*_1,22_ = 4.95; *P *≤* *.05). **P ≤* *.01 vs control by Aspin‐Welch test. (B) TAK‐063 (TAK, 0.1 mg/kg p.o.) and olanzapine (OLA, 2 mg/kg p.o.) were administered to rats 0.5 and 1.5 hours, respectively, before treatment with METH (0.5 mg/kg s.c.). Accumulated activity counts during 2 hours after METH treatment were calculated and indicated as the mean + SEM (n = 6 in control group, n = 7 in VEH + METH group, n = 6 in TAK + METH group, n = 7 in OLA + METH group, n = 6 in TAK + OLA + METH group). The two‐way ANOVA showed a significant interaction between OLA and TAK (*F*_1,22_ = 7.29; *P *≤* *.05). **P ≤* *.01 vs control by Aspin‐Welch test. (C) TAK‐063 (TAK, 0.1 mg/kg p.o.) and haloperidol (HAL, 0.3 mg/kg p.o.) were coadministered to rats 1.5 hours before treatment with MK‐801 (0.3 mg/kg s.c.). Accumulated activity counts during 2 hours after MK‐801 treatment were calculated and indicated as the mean + SEM (n = 5 in OLA + MK‐801 group, n = 6 in other groups). The two‐way ANOVA showed a significant interaction between HAL and TAK (*F*_1,20_ = 10.53; *P *≤* *.05). **P ≤* *.01 vs control by Aspin‐Welch test. (D) TAK‐063 (TAK, 0.1 mg/kg p.o.) and olanzapine (OLA, 3 mg/kg p.o.) were administered to rats 0.5 and 1.5 hours before treatment with MK‐801 (0.3 mg/kg s.c.), respectively. Accumulated activity counts during 2 hours after MK‐801 treatment were calculated and indicated as the mean + S.E.M. (n = 6). The two‐way ANOVA showed a significant interaction between TAK and OLA (*F*_1,19_ = 7.22; *P *≤* *.05). **P ≤* *.01 vs control by Aspin‐Welch test

TAK‐063 at 0.3 mg/kg p.o. (set as an efficacious dosage) produced potent antipsychotic‐like effects in METH‐ or MK‐801‐induced hyperactivity in rats (>50% suppression of hyperactivity).^15^, ^17^ Thus, augmented effects from combining 0.3 mg/kg TAK‐063 with current antipsychotics in these experimental paradigms would not be detected due to ceiling effects. To assess the effects of combined treatment with TAK‐063 and either haloperidol or olanzapine, we tested these drugs at subeffective doses on METH‐ or MK‐801‐induced hyperactivity in rats (0.1 mg/kg TAK‐063, 0.2 mg/kg haloperidol, and 2 mg/kg olanzapine in METH‐induced hyperactivity; 0.1 mg/kg TAK‐063, 0.3 mg/kg haloperidol, and 3 mg/kg olanzapine in MK‐801‐induced hyperactivity).^15^, ^17^ Combined treatment with TAK‐063 and either haloperidol or olanzapine at subeffective doses suppressed METH‐ or MK‐801‐induced hyperactivity in rats (Figure 4A‐D). The two‐way ANOVA analysis showed a significant interaction between TAK‐063 and either haloperidol or olanzapine (TAK‐063 and haloperidol in METH‐induced hyperactivity: *F*_1,22_ = 4.95; *P ≤ *.05; TAK‐063 and haloperidol in MK‐801‐induced hyperactivity: *F*_1,20_ = 10.53; *P ≤ *.05; TAK‐063 and olanzapine in METH‐induced hyperactivity: *F*_1,22_ = 7.29; *P ≤ *.05; TAK‐063 and olanzapine in MK‐801‐induced hyperactivity: *F*_1,19_ = 7.22; *P ≤ *.05; Figure 4A‐D). These results suggest that combined treatment with TAK‐063 and either haloperidol or olanzapine at subeffective doses produces augmented antipsychotic‐like effects in rats. Similar significant antipsychotic‐like effects were observed with combined treatment of TAK‐063 and aripiprazole (Figure S4).

### 3.4 Combined treatment with TAK‐063 and either haloperidol or olanzapine at subeffective doses has significant effects on MK‐801‐induced deficits on PPI in mice

**Figure 5:**
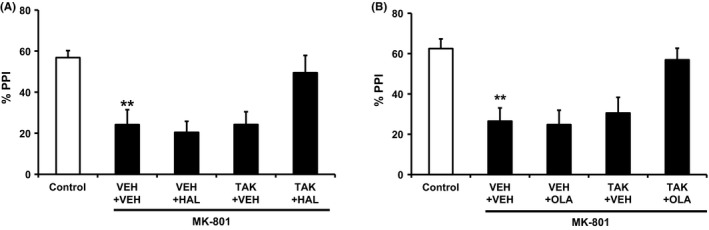
The effects of TAK‐063 combined with either haloperidol or olanzapine on MK‐801‐induced PPI deficits in mice. (A) Coadministration of TAK‐063 (TAK, 0.1 mg/kg p.o.) and haloperidol (HAL, 0.3 mg/kg p.o.), but not each drug alone, attenuated MK‐801‐induced PPI deficits in mice. Data (%PPI to the 82‐db prepulse) are presented as mean + SEM (n = 12 in control group, n = 10 in vehicle + MK‐801 group, n = 11 in other groups). **P ≤* *.01 vs control by Aspin‐Welch test. The two‐way ANOVA showed a significant interaction between TAK and HAL (*F*_1,39_ = 4.42; *P *≤* *.05). (B) Coadministration of TAK‐063 (TAK, 0.1 mg/kg p.o.) and olanzapine (OLA, 3 mg/kg p.o.), but not each drug alone, attenuated MK‐801‐induced PPI deficits in mice. Data (%PPI to the 82‐db prepulse) are presented as mean + S.E.M. (n = 9 in control group, n = 10 in vehicle + MK‐801 group, n = 11 in other groups). **P ≤* *.01 vs control by Aspin‐Welch test. The two‐way ANOVA showed a significant interaction between TAK and OLA (*F*_1,39_ = 4.20; *P *≤* *.05)

Disruption of PPI has been used as an animal model of schizophrenia‐like sensorimotor gating deficits and D_2_ antagonists and TAK‐063 at 0.3 mg/kg p.o. significantly ameliorated PPI deficits in mouse models.^15^, ^34^, ^35^ Like METH‐ or MK‐801‐induced hyperactivity, augmented effects on PPI deficits from combining 0.3 mg/kg TAK‐063 with current antipsychotics would not be detected due to ceiling effects. Thus, to assess the effects of combined treatment with TAK‐063 and either haloperidol or olanzapine, we used these drugs at subeffective doses in MK‐801‐induced PPI deficits in mice (0.1 mg/kg TAK‐063, 0.3 mg/kg haloperidol, and 3 mg/kg olanzapine). MK‐801 at 0.3 mg/kg s.c. significantly reduced the percentage of PPI in mice (*P ≤ *.01; Figure 5A and B). Coadministration of TAK‐063 and either haloperidol or olanzapine increased the percentage of PPI in MK‐801‐treated mice (Figure 5A and B). A two‐way ANOVA indicated an significant interaction between TAK‐063 and either haloperidol or olanzapine (TAK‐063 and haloperidol: *F*_1,39_ = 4.42; *P ≤ *.05; TAK‐063 and olanzapine: *F*_1,39_ = 4.20; *P ≤ *.05; Figure 5A and B). Similar significant effects on PPI in MK‐801‐treated mice were observed with combined treatment TAK‐063 and aripiprazole (Figure S4). These results further support that coadministration of TAK‐063 and current antipsychotics could produce augmented antipsychotic‐like effects in rodents.

### 3.5 TAK‐063 at 0.1 mg/kg did not exacerbate the effects of haloperidol or olanzapine on plasma prolactin and cataleptic response in rats

**Figure 6:**
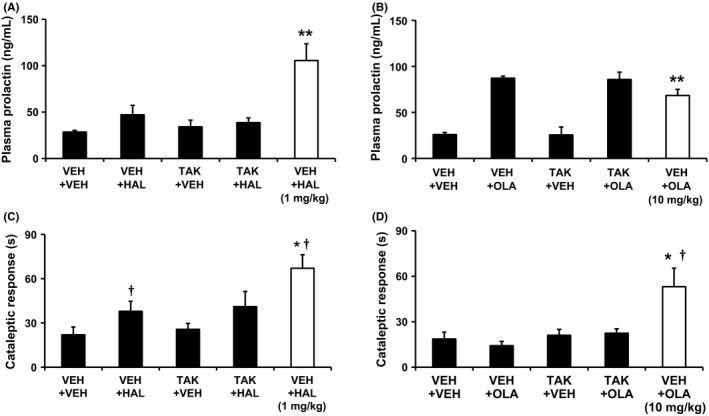
The effects of TAK‐063 combined with either haloperidol or olanzapine on plasma prolactin level and cataleptic response in rats. TAK‐063 (TAK, 0.1 mg/kg p.o.) and either haloperidol (A) (HAL, 0.3 mg/kg p.o.) or olanzapine (B) (OLA, 3 mg/kg p.o.) were coadministered to male SD rats, and then blood was collected from their tail veins 1.5 hours after administration. Prolactin concentration in the plasma was determined by ELISA. Data are presented as mean + SEM (n = 6). **P ≤* *.01 vs vehicle + vehicle treatment group by Aspin‐Welch test. The two‐way ANOVA showed no interaction between TAK and either HAL or OLA. Duration of cataleptic response was measured by bar test 4 hours after coadministration of TAK‐063 (0.1 mg/kg p.o.) and either HAL (C) (0.3 mg/kg p.o.) or OLA (D) (3 mg/kg p.o.). HAL at 1 mg/kg or OLA at 10 mg/kg was used as a positive control. Data are represented as mean + SEM (n = 6). ^†^Appearance of animals in a cataleptic position over 90 seconds (cutoff value). *P ≤* *.05 vs vehicle + vehicle treatment group by Aspin‐Welch test. The two‐way ANOVA showed no interaction between TAK and either HAL or OLA

Current antipsychotics cause hyperprolactinemia by D_2_ receptor antagonism in the pituitary gland, and EPS such as Parkinsonism probably due to excessive activation of the indirect pathway.^9^, ^36^, ^37^ The combined effects of TAK‐063 and either haloperidol or olanzapine on plasma prolactin levels and potential for inducing Parkinsonism were evaluated in rats. To assess the benefit of a reduction in the risk for hyperprolactinemia and inducing Parkinsonism while maintaining antipsychotic‐like effects, we used the doses of the test compounds producing significant antipsychotic‐like effects on MK‐801‐induced hyperactivity in rats (Figure 4C and D). Coadministration of TAK‐063 at 0.1 mg/kg p.o. and haloperidol at 0.3 mg/kg p.o. did not affect plasma prolactin levels, whereas haloperidol at 1 mg/kg p.o. alone significantly increased plasma prolactin levels (*P ≤ *.01; Figure 6A). When 0.1 mg/kg TAK‐063 was combined with 3 mg/kg olanzapine, the effect of TAK‐063 on olanzapine‐induced plasma prolactin increase was not determined because the maximum effect was observed with olanzapine even at 3 mg/kg alone; olanzapine at 3 and 10 mg/kg increased plasma prolactin to a similar extent (Figure 6B). The cataleptogenic activities of TAK‐063 dosed in combination with either haloperidol or olanzapine were evaluated in rats to assess the potential to produce Parkinsonism with combination therapy. TAK‐063 at 0.1 mg/kg did not affect haloperidol at 0.3 mg/kg‐ or olanzapine at 3 mg/kg‐induced cataleptic response in rats (Figure 6C and D). The two‐way ANOVA analysis showed no interactions between TAK‐063 and either haloperidol or olanzapine (*P > *.05; Figure 6C and D).

## 4 DISCUSSION

Both direct and indirect pathways have competing effects on antipsychotic‐like activities and motor functions in rodents.^15^, ^16^, ^17^ Compared to other PDE10A inhibitors, the balanced activation of the direct and indirect pathway MSNs by TAK‐063 may represent a novel therapeutic approach to treat patients with schizophrenia, either alone or in combination with other D_2_ antagonist antipsychotics. In this study, we evaluated combined effects of TAK‐063 with current antipsychotics haloperidol or olanzapine on the activation of MSN pathways, antipsychotic‐like effects, plasma prolactin levels, and cataleptic responses in rodents.

Given the high level of expression of PDE10A in both direct and indirect pathway MSNs, PDE10A inhibition and resulting elevation of cAMP and cGMP is expected to increase neural activities in both types of MSNs. To confirm this idea, we evaluated the effects of PDE10A inhibition by TAK‐063 on NMDA receptor‐mediated synaptic responses in each type of MSNs using rat corticostriatal brain slices. Corticostriatal responses in direct and indirect MSNs are radically different and electrophysiological properties allow us to discriminate between them in rat corticostriatal brain slices.^20^ TAK‐063 enhanced NMDA‐receptor‐mediated synaptic responses in both direct and indirect pathway MSNs to a similar extent. In line with these results, TAK‐063 increased the number of MSNs expressing *c‐fos* mRNA in both direct and indirect MSNs of the striatal complex in rats.^38^ To further investigate the activation pattern of each MSN pathway by the combination of TAK‐063 and either haloperidol or olanzapine in vivo, gene expression changes in pathway‐specific markers (substance P and enkephalin mRNAs) were evaluated in the rat striatum. Combined treatment with TAK‐063 and either haloperidol or olanzapine showed an additive effect on the activation of the indirect pathway MSNs, but not on the activation of the direct pathway MSNs. Combined treatment with TAK‐063 and these antipsychotics increased pGluR1 in the rat striatum, which was inconsistent result with an additive effect on expression of enkephalin mRNAs. Although the precise reasons for this inconsistency remain unclear, a difference in sensitivity of responsiveness of these two biochemical measurements to activation of cAMP and cGMP signaling is possibly considered: responsiveness of induction of enkephalin mRNA by activation of cAMP and cGMP signaling could be lower than that of upregulation of pGluR1. In fact, a PDE10A selective inhibitor MP‐10 at 3 mg/kg produced an approximately 5‐fold increase in pGluR1, whereas it only produced 1.3‐fold increase in enkephalin mRNA in the striatum.^26^ Detailed studies such as a comprehensive gene expression analysis, but not measurements of only some specific targets, in each MSN pathway would be worth better understanding of an augmented effect on the activity in MSNs by the combination of TAK‐063 and a D_2_ antagonist.

Combined treatment with TAK‐063 and either haloperidol or olanzapine at subeffective doses showed the potential of augmented antipsychotic‐like effects as assessed by METH‐ or MK‐801‐induced hyperactivity in rats and MK‐801‐induced PPI deficits in mice. A limitation of these behavioral assays is that only a single dose of TAK‐063 and haloperidol or olanzapine has been explored. Even though statistically significant interaction was observed, experiments using multiple doses will be ideal in order to fully characterize the effects by combined treatment with TAK‐063 and current antipsychotics. However, a ceiling effect will be observed when higher doses of TAK‐063 and/or current antipsychotics are used in these behavioral assays. In fact, TAK‐063 at 0.1 and 0.3 mg/kg showed 17% and 95% inhibition of MK‐801‐induced hyperactivity in rats.^17^ Thus, to further support augmented antipsychotic‐like effects observed in this study, other behavioral assays which can evaluate pharmacological effects over a wider dose range should be required for a future multiple‐dose study.

Some of the current antipsychotics cause hyperprolactinemia owing to their D_2_ receptor antagonism in the pituitary gland.^9^, ^36^ TAK‐063 does not cause hyperprolactinemia in rats as PDE10A expression is low in the pituitary gland.^13^, ^39^ Thus, combination of TAK‐063 with haloperidol at subeffective doses did not exacerbate the effects on plasma prolactin in rats.

In addition to extrapyramidal side effects and hyperprolactinemia, atypical antipsychotics such as olanzapine, clozapine, and quetiapine have metabolic side effects that include weight gain and diabetes.^40^, ^41^ Data from the Clinical Antipsychotic Trials of Intervention Effectiveness study have also indicated that the general prevalence of metabolic syndrome is twice as much in patients with schizophrenia.^42^ Interaction with multiple receptors other than D_2_ receptor, such as histamine H1 receptor, is speculated to contribute to these metabolic side effects.^9^ Interestingly, PDE10A inhibitors are reported to have beneficial effects on metabolic dysfunctions: body weight loss, reduced adiposity, and increased whole‐body energy expenditure in mice with diet‐induced obesity.^43^, ^44^

In this study, the combination of TAK‐063 and current antipsychotics produced the augmentation of the activation of the indirect pathway MSNs while maintaining activation of the direct pathway MSNs. TAK‐063 significantly produces potent antipsychotic‐like effects with haloperidol or olanzapine at subeffective doses without effects on plasma prolactin levels and cataleptic responses in rodents. The use of more than one antipsychotic for the treatment of patients with schizophrenia is a common clinical practice that attempts to increase efficacy and reduce side effects by dose reduction in each antipsychotic^45^, ^46^, ^47^, ^48^; however, clinical evidence for the benefits of cotreatment with current antipsychotics has not yet been clearly established.^49^ Treatment with combinations of antipsychotics with the same mechanism of action is one possible explanation for the lack of clear therapeutic benefit. Further preclinical and clinical studies will be needed to investigate the possibility of combination therapy with TAK‐063 and current antipsychotics. TAK‐063 is currently in clinical development.

## DISCLOSURES

K.S., A.H., H.S., and H.K. are employees of Takeda Pharmaceutical Company Limited. C.C., A.U., and M.C. are employees of Aptuit Inc.

## AUTHORS’ CONTRIBUTIONS

*Participated in research design:* K. Suzuki, Corsi, Kimura. *Conducted experiments:* K. Suzuki, Harada, H. Suzuki, Capuani, Ugolini. *Performed data analysis:* K. Suzuki, Harada, H. Suzuki, Capuani, Ugolini, Corsi, Kimura. *Wrote or contributed to the writing of the manuscript:* K. Suzuki, Kimura.
