# Supplementary material for: Combined treatment with a selective PDE10A inhibitor TAK‐063 and either haloperidol or olanzapine at subeffective doses produces potent antipsychotic‐like effects without affecting plasma prolactin levels and cataleptic responses in rodents
Source: Pharmacol Res Perspect. 2017 Dec 18;6(1):e00372. doi: 10.1002/prp2.372 (PMC5817828; doi:10.1002/prp2.372)
Supplement: Supplementary file 1 [file PRP2-6-e00372-s001.pdf]

Combined treatment with a selective PDE10A inhibitor TAK-063 and either haloperidol or olanzapine at subeffective doses produces potent antipsychotic-like effects without affecting plasma prolactin levels and cataleptic responses in rodents

Kazunori Suzuki, Akina Harada, Hirobumi Suzuki, Clizia Capuani, Annarosa Ugolini, Mauro Corsi, and Haruhide Kimura

CNS Drug Discovery Unit, Research, Takeda Pharmaceutical Company Limited, Fujisawa, Kanagawa, Japan (K.S., A.H., H.S., H.K.); Center of Drug Design & Discovery, Aptuit Inc., Verona, Italy (C.C., A.U., M.C.)

Supplemental Fig. 1

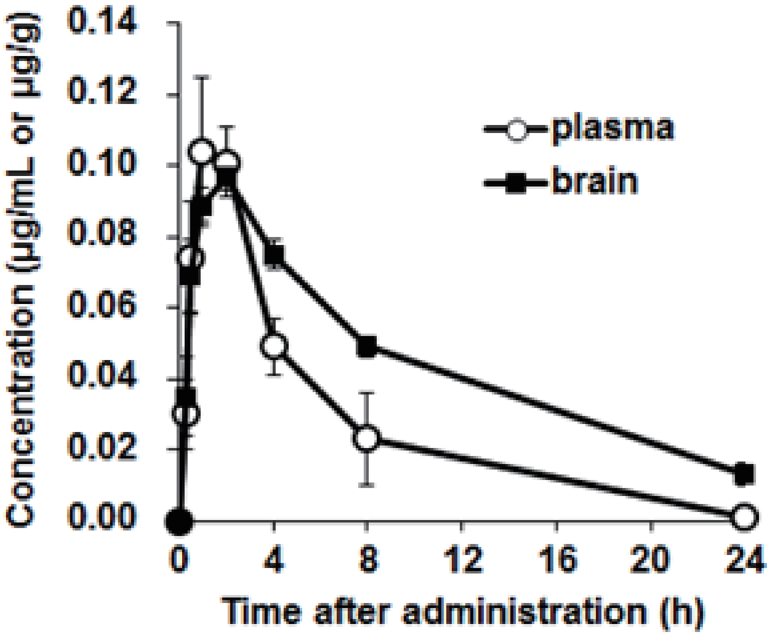

Supplemental Fig. 1. Plasma concentration of TAK-063 in ICR mice. Mice were administered TAK-063 at 0.3 mg/kg p.o. and sacrificed at the indicated time to obtain plasma samples. Data are represented as mean ± S.D. (n = 3 per time point). Cmax in plasma and brain is 0.104 μg/mL and 0.097 μg/g, respectively. Tmax in plasma and brain is 1.00 h and 2.00 h, respectively. AUC_{0-24h} in plasma and brain is 0.650 μg·h/mL and 1.066 μg·h/g, respectively.

Materials and Methods for Supplemental Figure 1

Measurement of plasma concentration of TAK-063. Male ICR mice were supplied by supplied by CLEA Japan, Inc. (Tokyo, Japan). The mice were housed in a light-controlled room (12-h light/dark cycle, with lights on at 0700 hours). After an acclimation period of at least 1 week, mice were used at 7 weeks of age. The care and use of the animals and the experimental protocols were approved by the Institutional Animal Care and Use Committee of Takeda Pharmaceutical Company Limited. TAK-063 was suspended in 0.5% (w/v) methylcellulose in distilled water using an ultrasonic sonicator. TAK-063 (0.3 mg/kg) was orally administered to mice with a dose volume of 20 mL/kg. Mice were decapitated 0.25, 0.5, 1, 2, 4, 8, and 24 h after administration. Trunk blood was collected into 1.5-mL centrifuge tubes. Blood samples were immediately mixed with EDTA and then centrifuged at 15000 rpm for 15 min at 4ºC. The supernatants were collected in another tube as plasma. TAK-063 concentration was measured in aliquots of mouse plasma which were mixed well with acetonitrile containing an analog of TAK-063 as internal standards and then centrifuged. TAK-063 concentrations were measured in aliquots of mouse plasma which was mixed well with acetonitrile containing an analog of TAK-063 as internal standards and then centrifuged. The supernatants were diluted with solvents for LC-MS/MS analysis (mobile phase A:

10 mM ammonium formate/formic acid [100/0.2, v/v], mobile phase B: acetonitrile/formic acid [100/0.2, v/v]). The diluted solutions (1 μL) were injected into an LC-MS/MS (API5000, AB Sciex, Foster City, CA) that was equipped with Shimadzu Shim-pack XR-ODS (2.2 μm, 2.0 × 30 mm) maintained at 50°C. The chromatographic separation was performed with a gradient elution at a flow rate of 0.7 mL/min. The LC time program was the following: Mobile phase B was held at 5% for 0.1 min, and increased linearly to 95% for 0.1 min. After B was held at 95% for another 0.8 min, it was brought back to 5% B in 0.01 min, followed by re-equilibration for 0.59 min. The total cycle time for one injection was 1.6 min. TAK-063 was detected using a multiple reaction monitoring mode using the transition: TAK-063 m/z 429.30 → 170.40, internal standard m/z 434.10 → 175.40. Analyst softwareTM (version 1.4.2) was used for data acquisition and processing.

Supplemental Fig. 2

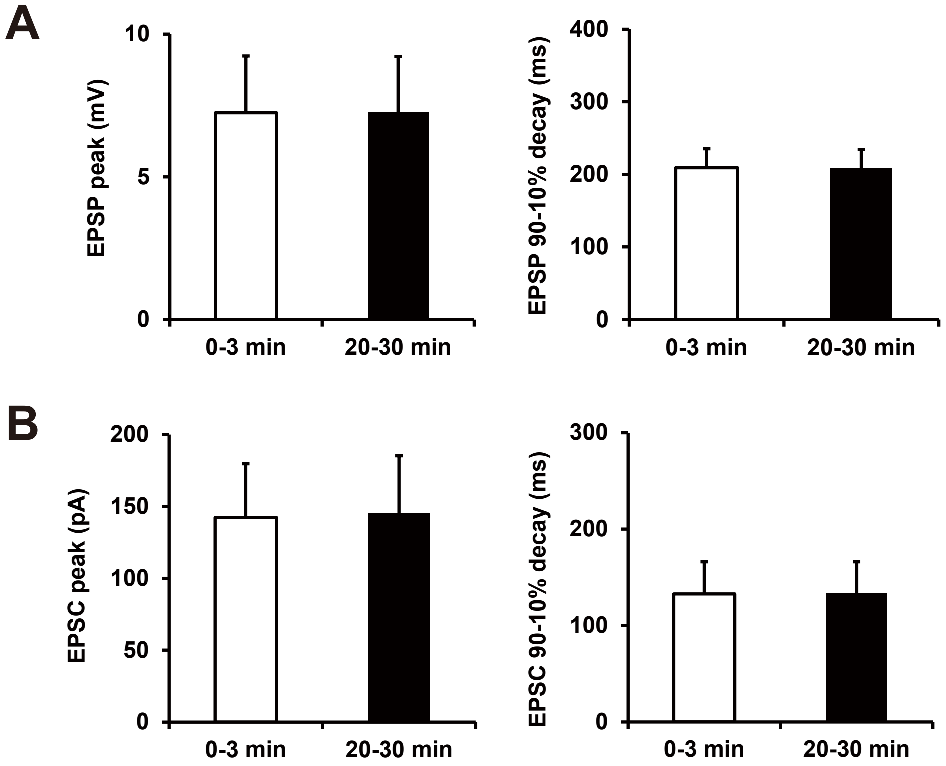

Supplemental Fig. 2. The effect of vehicle on NMDA receptor-mediated synaptic responses in MSNs in rat corticostriatal brain slices. No significant effect of vehicle (0.1% dimethyl sulfoxide) application was observed on peak amplitudes and decay times of NMDAR EPSPs (A) and EPSCs (B) in MSNs. White bar indicates the average of peak amplitudes and decay times of EPSPs and EPSCs during the first 3 min after application of vehicle. Black bar indicates the average of peak amplitudes and decay times of EPSPs and EPSCs during 3 min between 20-30 min after application of vehicle. Data are represented as mean + S.E.M. (n = 10-12).

Supplemental Fig. 3

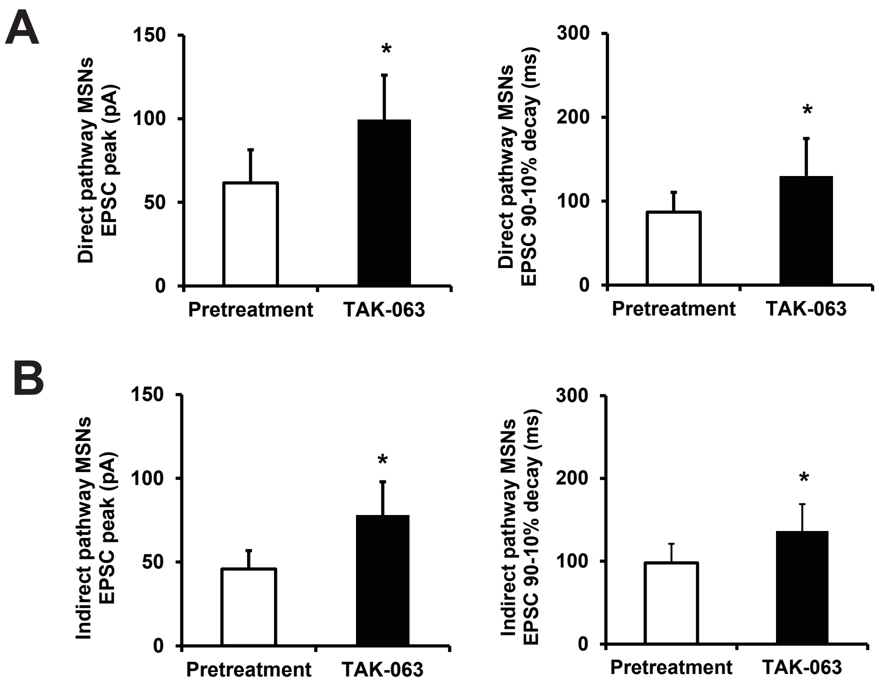

Supplemental Fig. 3. The effects of TAK-063 on NMDA receptor-mediated synaptic responses in each type of MSNs in rat corticostriatal brain slices. TAK-063 at 1 µM significantly increased peak amplitudes and decay times of NMDAR EPSCs in direct pathway MSNs (A) and indirect pathway MSNs (B). The average of peak amplitudes and decay times during the 3 min before and after the 20 min of compound application was calculated. Data are represented as mean + S.E.M. (n = 7). *P ≤ 0.05 versus control (pretreatment) by Wilcoxon matched-pairs signed rank test.

Supplemental Fig. 4

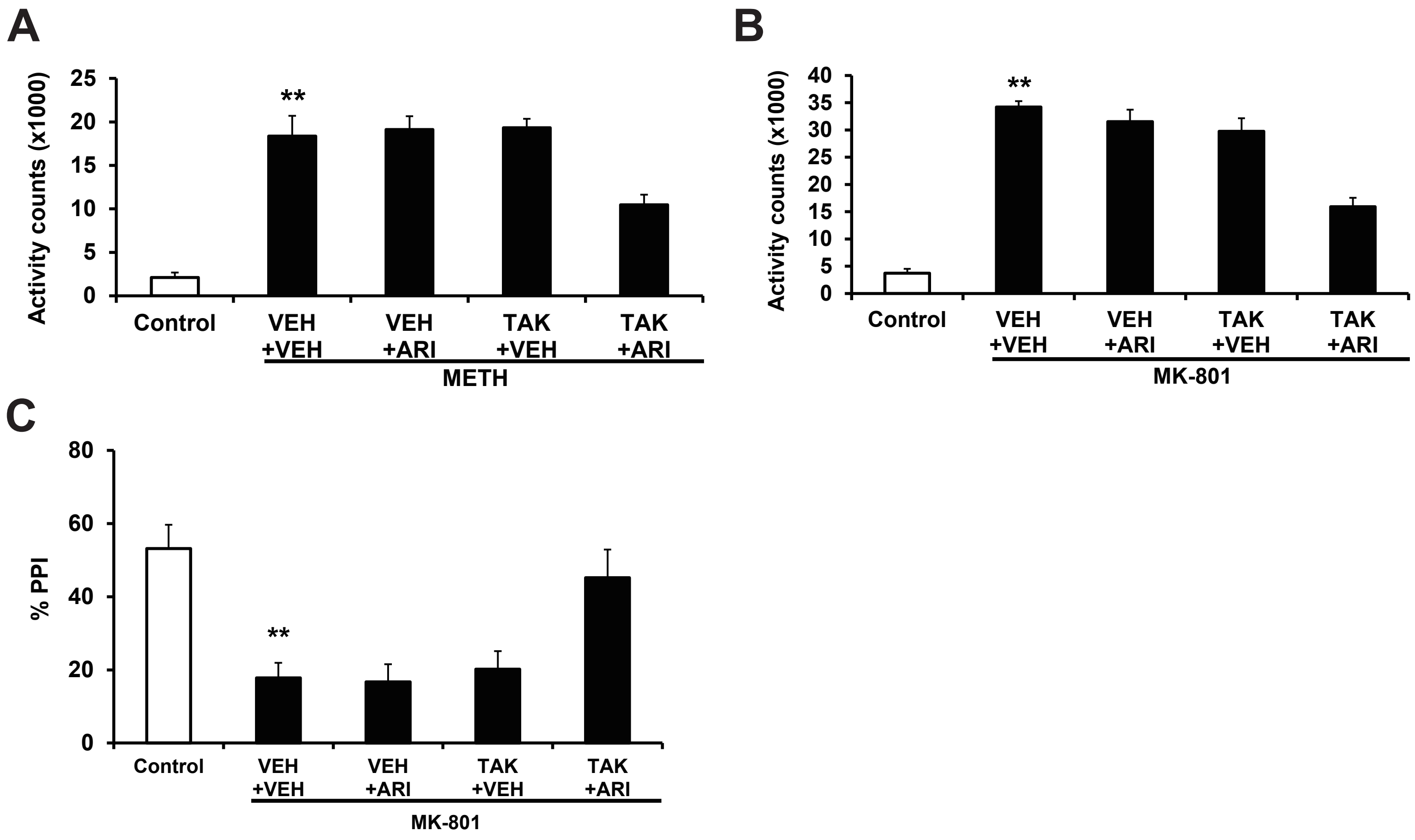

Supplemental Fig. 4. The effects of TAK-063 combined with aripiprazole on METH- or MK-801–induced hyperactivity in rats and MK-801–induced deficits in PPI in mice. (A) TAK-063 (TAK, 0.1 mg/kg p.o.) and aripiprazole (ARI, 3 mg/kg p.o.) were coadministered to rats 1.5 h before treatment with METH (0.5 mg/kg s.c.). Accumulated activity counts during 2 h after METH treatment were calculated and indicated as the mean + S.E.M. (n = 6 in control group, n = 6 in vehicle (VEH) + METH group, n = 7 in TAK + METH group, n = 6 in ARI + METH group, n = 7 in TAK + ARI + METH group). The two-way ANOVA showed a significant interaction between ARI and TAK ($F_{1,22}$ = 9.84; $P \leq 0.05$). **$P \leq 0.01$ versus control by Aspin–Welch test. (B) TAK-063 (TAK, 0.1 mg/kg p.o.) and aripiprazole (ARI, 10 mg/kg p.o.) were administered to rats 1.5 h before treatment with MK-801 (0.3 mg/kg s.c.). Accumulated activity counts during 2 h after MK-801 treatment were calculated and indicated as the mean + S.E.M. (n = 6 in control group, n = 6 in vehicle (VEH) + MK-801 group, n = 7 in TAK + MK-801 group, n = 6 in ARI + MK-801 group, n = 7 in TAK + ARI + MK-801 group). The two-way ANOVA showed a significant interaction between ARI and TAK ($F_{1,22}$ = 8.75; $P \leq 0.05$). **$P \leq 0.01$ versus control by Aspin–Welch test. (C) Coadministration of TAK-063 (TAK, 0.1 mg/kg p.o.) and aripiprazole (ARI, 10 mg/kg p.o.), but not each drug alone, attenuated MK-801–induced PPI deficits in mice. Data (%PPI to the 82-db prepulse) are presented as mean + S.E.M. (n = 12 in control group, n = 10 in vehicle + MK-801 group, n = 11 in other groups). **$P \leq 0.01$ versus control by Aspin-Welch test. The two-way ANOVA showed a significant interaction between TAK and ARI ($F_{1,41}$ = 5.60; $P \leq 0.05$).
